# Obesity paradox in cognitive function: a longitudinal study of BMI and cognitive impairment in older adult Chinese population

**DOI:** 10.3389/fnagi.2025.1543501

**Published:** 2025-05-09

**Authors:** Jing Chen, Jun Zhang, Hao Wang, Hongliang Zhu, Jialin Fu, Chuanwei Li, Qingrong Zhang, Xingzhi Xia, He Ma, Junjun Liu, Xiangdong Du

**Affiliations:** ^1^Suzhou Guangji Hospital, The Affiliated Guangji Hospital of Soochow University, Suzhou, China; ^2^Suzhou Medical College of Soochow University, Suzhou, China; ^3^Xuzhou Medical University, Xuzhou, China; ^4^Nanjing Meishan Hospital, Nanjing, China

**Keywords:** body mass index, cognitive impairment, obesity paradox, older adult population, non-linear association

## Abstract

**Objective:**

This study endeavored to investigate the correlation between body mass index (BMI) and cognitive impairment in the demographic of Chinese individuals who are 60 years of age and above.

**Methods:**

We selected data from China Health and Retirement Longitudinal Study (CHARLS) 2015–2018 and 2,942 subjects aged ≥60 years were included. The Mini-Mental State Examination (MMSE) was used to assess cognitive impairment. BMI was examined in two forms: as a continuous variable and was stratified into tertiles. Analysis was conducted using both univariate and multivariate logistic regression. Non-linear relationships were analyzed using curve fitting and segmented logistic regression.

**Results:**

During the study, 600 out of 2,942 subjects (20.4%) experienced cognitive impairment. In fully adjusted models, each unit increase in BMI was related to a 4% decrease in the odds of cognitive impairment (OR: 0.96, 95% CI: 0.93–0.99, *p* = 0.008). There was a noticeable protective effect from the highest BMI tertile in comparison to the lowest tertile (OR: 0.64, 95% CI: 0.50–0.83, *p* < 0.001). Non-linear analysis revealed an inflection point at BMI of 26.60 kg/m^2^, with a significant inverse relationship below this point (OR: 0.96, 95% CI: 0.93–0.99, *p* = 0.008) and no substantial association above it.

**Conclusion:**

This study provides evidence supporting the “obesity paradox” in the cognitive function of older adult Chinese population. Higher BMI is linked to lower cognitive impairment risk, especially among overweight persons. These findings indicate a complex and non-linear link between BMI and cognitive health among older adult adults, emphasizing the importance of tailored strategies for weight management in this population.

## 1 Introduction

Cognitive impairment is a progressive deterioration of cognitive abilities, often associated with aging and considered a precursor to dementia (Petersen, 2016). It is highly prevalent among older adult adults, with the rates ranging from 10 to 20% in populations aged 65 and above (Plassman et al., 2008). For instance, a study by Mantzorou et al. (2020) reported that 34.4% of 2,093 adults aged ≥65 years from seven Greek cities experienced cognitive impairment. In China, cognitive impairment among older adults has been exacerbated in recent years by population aging, urbanization, population mobility, challenges in primary health care, and issues related to social awareness and stigma (Wang et al., 2025; Zhang et al., 2023). Wang et al. (2022) found that the prevalence of suspected dementia was 26.8% among 773 adults aged ≥65 years in primary health care clinics in Wuhan, China. Cognitive impairment is closely related to numerous adverse outcomes in older adult adults, including poor quality of life, loss of independence, and even death (Deary et al., 2009). Given its significant impact, understanding the mechanisms underlying cognitive impairment in older adult adults is crucial. This knowledge can help develop effective prevention and intervention strategies for this population. There are several risk factors identified for the cognitive impairment in older adult adults, including age, education level, obesity, genetic predisposition, cardiovascular health, and lifestyle factors (Livingston et al., 2017). Among them, BMI has been noted to be a potentially modifiable determinant related to the cognitive impairment in older adult adults. It is calculated from an individual’s height and weight, serving as a simple measure of body fat and has been widely used in clinical settings and epidemiological studies as an indicator of overall health status (World Health Organization, 2000).

The connection between BMI and cognitive impairment among the older adult has been the subject in numerous studies, with varying results. A variety of studies have revealed BMI is positively related to the cognitive impairment. For example, in a cohort of 8,534 Swedish twins aged 65 and older adult, Xu et al. (2011) observed that increased BMI was associated with more rapid cognitive impairment during middle age. Singh-Manoux et al. (2012) found that obesity (BMI > 30 kg/m^2^) was linked to lower cognitive function among 6,401 British individuals aged 50–63 years. Similarly, Liu et al. (2019) reported in a cross-sectional study of 1,037 Chinese adults aged ≥65 years that elevated BMI was associated with worse cognitive performance. These associations have been attributed to some mechanisms, including inflammation, vascular dysfunction, and altered brain structure (Bischof and Park, 2015). Nevertheless, there are inconsistent results regarding BMI’s connection to cognitive impairment. A study involving 3,885 American adults aged 65 and above did not reveal any considerable link between BMI and cognitive performance (Sturman et al., 2008). Furthermore, certain research has indicated a U-shaped pattern linking BMI to cognitive performance, suggesting that both underweight and overweight individuals may be at increased risk for cognitive impairment (Fitzpatrick et al., 2009). Of interest, a few studies have even reported BMI is negatively related to the cognitive function. In a study of Kim et al. (2016), higher BMI correlated with a reduced rate of cognitive impairment in 1,324 Korean adults aged 65 and above. Hou et al. (2019) found that being overweight exerted a protective effect on cognitive function in 1,100 Chinese individuals aged 60–98 years, and Vidyanti et al. (2020) reported similar findings in a study on 143 older adult individuals in Yogyakarta.

This inconsistency in these findings has led to the concept of “BMI paradox” in cognitive impairment, where overweight or obesity appears to be protective against cognitive impairment in some older adult populations (Qizilbash et al., 2015). For example, studies have revealed that individuals with a higher BMI (≥25 kg/m^2^) are less likely to develop cognitive impairment (Xiang and An, 2015; Suemoto et al., 2015; Aslan et al., 2015; Anstey et al., 2011; Atti et al., 2008; Hughes et al., 2009). These inconsistencies may, at least in part, be attributed to variations in study populations, methodologies, and the complex, potentially non-linear relationship between BMI and cognitive function. These conflicting findings highlight the necessity of rigorously designed cohort studies to clarify the precise relationship between BMI and cognitive health. To elucidate the complex relationship between BMI and cognitive impairment, especially in diverse and aging populations, large-scale longitudinal studies are indispensable.

CHARLS is a national longitudinal survey, characterized by its large sample size, diverse population, and longitudinal design. It serves as an ideal dataset for investigating the relationship of BMI with cognitive impairment in the older adult, offering an excellent opportunity to address this research gap. CHARLS investigated individuals aged 45 and up, and provided entire data on health, economic, and social factors (Zhao et al., 2014). Utilizing the data from the CHARLS 2015–2018 cohort, our study endeavored to investigate the relationship between BMI and cognitive health in Chinese adults aged ≥60 years. This research is innovative in its use of a large, representative sample of the Chinese older adult population and its longitudinal design, which allows for the assessment of BMI’s impact on cognitive impairment over time. Our findings will contribute to the understanding of the BMI paradox and offer key insights on the intricate correlation between BMI and cognitive health among the older adult in China.

## 2 Methods

### 2.1 Design, setting, and study population

The CHARLS is a large-scale long-term panel survey project hosted by the National School of Development at Peking University and implemented by the Institute of Social Science Survey at Peking University. The project recruited participants from 450 communities in 150 counties/districts across 28 provinces in mainland China, excluding Tibet. CHARLS provides a comprehensive longitudinal dataset for investigating the health and well-being of China’s middle-aged and older adult people. In CHARLS, data are publicly accessible to researchers who signed a data usage agreement and provided basic information. A stratified, multi-stage sampling framework with probabilities scaled to the size of the population was employed to ensure that the sample was representative of the participants. In each randomly chosen household, one person aged ≥45 years was selected as the lead respondent, and both the respondent and their spouse were interviewed. Given the complexity of CHARLS, which covers multiple aspects of personal life, trained investigators conducted in-person interviews with computer-assisted personal interviewing (CAPI), with a response rate of 80.5% from the households that were eligible (Zhao et al., 2014).

The initial CHARLS visit was completed in 2011–2012 with 17,708 participants, followed by three biennial follow-up visits (2013–2014, 2015–2016, and 2017–2018). Trained investigators collected different information, including socioeconomic status, chronic diseases, physical function, family structure, healthcare utilization, and insurance participation. Above information is accessible on the CHARLS website^1^ (Zhao et al., 2014).

The Biomedical Ethics Review Board of Peking University approved the research protocol (IRB00001052-11015), and all participants offered their written consent formally.

**Figure 1:**
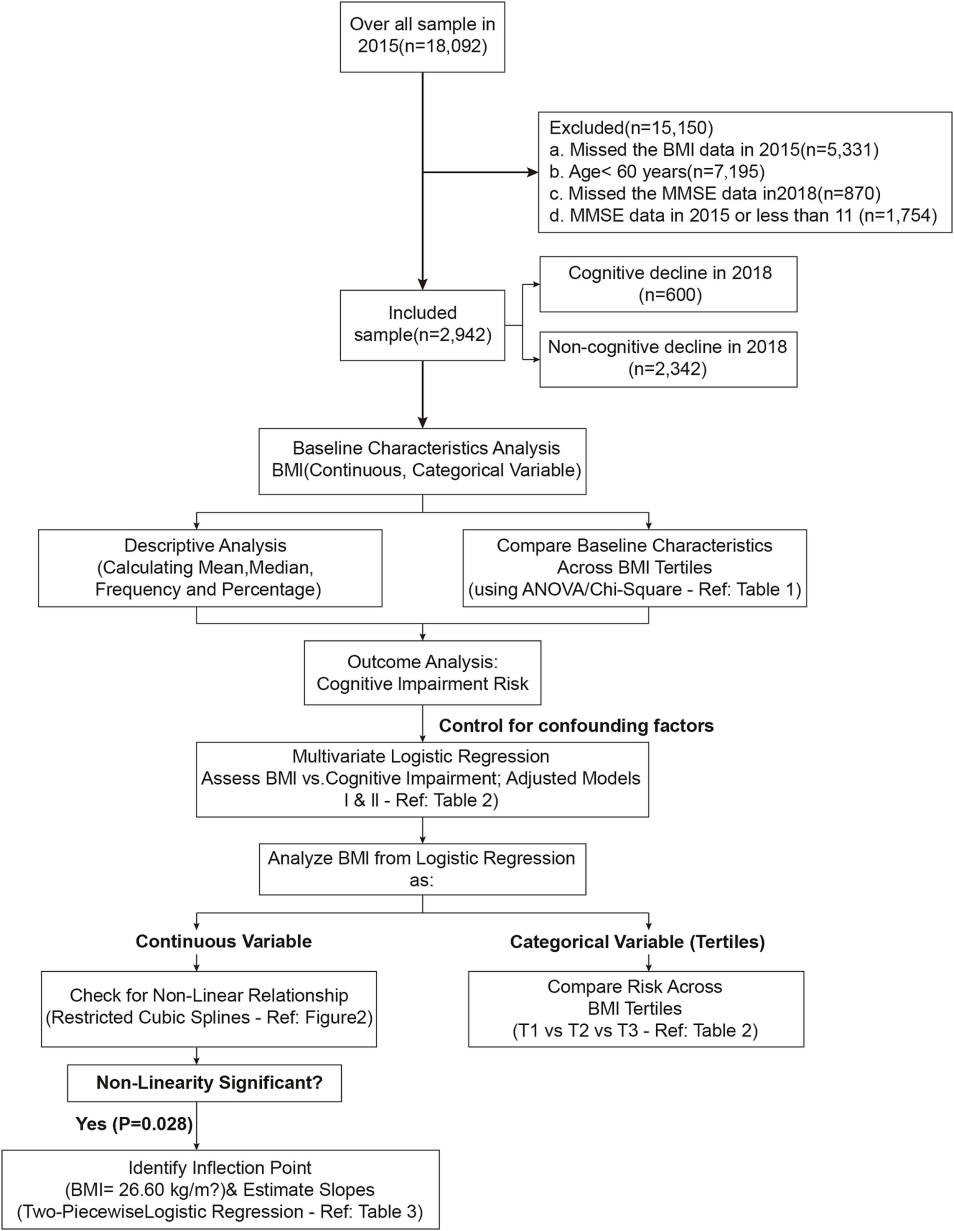
Flow chart of this study.

In the present study, the 2015–2018 follow-up data from CHARLS were used, involving 18,092 participants. The participants were excluded according to following criteria: 5,331 subjects without information about BMI; 7,195 subjects younger than 60 years; 1,754 subjects without baseline MMSE assessments or with MMSE scores less than 11; and 870 subjects without subsequent MMSE assessments in 2018. Finally, 2,942 subjects were analyzed in this study. Figure 1 illustrates the study flow chart.

### 2.2 Cognitive function assessment

Within this research, the primary outcome measured was cognitive function in 2018. In the CHARLS, cognitive function was assessed with MMSE (Chinese version), in which two dimensions were assessed: episodic memory and mental intactness (Chan et al., 2023; Ren et al., 2021). Episodic memory was tested by immediate and delayed recall of a ten-word list (Sha et al., 2018). The researcher pronounced ten distinct Chinese words aloud, and asked the subjects recollect the words without delay (immediate memory) and again after a 5-min interval (delayed memory). Each correctly recalled word was scored as one point. The final episodic memory score was calculated by adding the scores from immediate and delayed recall tests (0 to 20) (Liu et al., 2021). The Telephone Interview of Cognitive Status (TICS) and a visuospatial ability test were utilized to assess mental intactness (Wei et al., 2018). The TICS assessment included items such as identifying the current date (year, month, and day), the season, the weekday, and perform a 100 minus 7 combo operation up to 5 times. A figure drawing assignment was used to measure visuospatial ability. The investigator presented the subject a picture and then asked the subject to recreate it. Each right answer was worth one point (else 0). The mental intactness was evaluated by adding the TICS and visuospatial ability scores (0–11). The global cognition score, ranging from 0 to 31, was derived by summing the scores of episodic memory and mental intactness. Higher scores indicate better cognitive healthy. In the present study, a global cognition score lower than 11 was used to define cognitive impairment (Crimmins et al., 2011; Zhou et al., 2020), while scores of 11 or higher were classified as normal cognitive function.

### 2.3 Blood sample collection

Results from blood examination in the present study were obtained from the survey in 2015. After fasting overnight, a venous blood sample was collected at local Centers for Disease Control (CDC) or other medical facility and was immediately centrifuged. The plasma was harvested and preserved at −20°C. The samples were shipped to Beijing’s CDC within a fortnight and subsequently stored at −80°C for further biochemical analyses, including serum creatinine, glucose, and blood lipids.

The blood samples were analyzed at Capital Medical University’s Youanmen Center for Clinical Laboratory. Serum creatinine concentrations were assessed employing the rate-blanked, modified Jaffe technique. Triglycerides (TG), total cholesterol (TC), low-density lipoprotein cholesterol (LDL-C), and high-density lipoprotein cholesterol (HDL-C) were all identified using the enzymatic colorimetric assay.

### 2.4 Demographic characteristics and other covariates

Demographic and socioeconomic data were collected, including participants’ age, gender, marital status, educational attainment (categorized as no formal education, junior school, senior school, or college and above), and residential location (rural or urban). Health behavior information included current smoking status and current alcohol consumption. Additionally, health condition information included self-reported diabetes mellitus, hypertension, and dyslipidemia.

In this study, BMI was calculated by measuring the participants’ barefoot standing height (m) and body weight (kg) while wearing light clothing. Both measurements were recorded to one decimal place. BMI is defined as the weight in kilograms divided by the square of the height in meters (kg/m^2^). Blood pressure, including diastolic (DBP) and systolic (SBP), was measured at the left brachial artery while participants were seated calmly. Blood pressure was tested thrice with an interval of 45 s between two measurements, followed by the calculation of averages.

### 2.5 Statistical analysis

Categorical variables are presented as frequencies or percentages, while continuous variables are represented by mean ± standard deviation (SD). Baseline characteristics were analyzed across BMI tertiles using the Chi-square test for categorical variables and one-way analysis of variance (ANOVA) for continuous variables. Logistic regression with multiple covariates was applied to investigate the correlation between BMI and cognitive impairment, considering BMI in both continuous and categorical (tertile) forms. There were three models in this study: Unadjusted Model was not adjusted; Model I was adjusted for marital status, age, sex, and residence; Model II was further adjusted for drinking, hypertension, diabetes mellitus, dyslipidemia, creatinine, LDL cholesterol, HDL cholesterol, total cholesterol, smoking, and BMI. These variables were chosen for inclusion due to their correlations with cognitive impairment (*p* < 0.10 in single-variable analyses) or their impact on altering the effect size estimate by more than 10% upon their incorporation into the model [22, 23]. Although some of these variables did not reach the more stringent statistical significance threshold of *p* < 0.05 in univariate analyses, they were included to comprehensively account for potential confounding factors that may influence cognitive outcomes. The variance inflation factor (VIF) was calculated for each covariate to avoid multicollinearity. Variables with VIF > 5.0 were considered to have high collinearity and then excluded from the final model. The median BMI value for each tertile was incorporated as a continuous variable into the models to assess linear trends. Cubic spline regression with restriction was applied to investigate the possible nonlinear relationship between BMI and cognitive impairment, with knots set at the 10th, 50th, and 90th percentiles of BMI distribution. If a non-linear association was detected, a two-segmented logistic regression model was applied to identify the inflection point of BMI using a recursive algorithm. The inflection point was determined based on the maximum likelihood estimation.

Statistical analyses were performed using R software (version 4.0.3) and EmpowerStats. ‘STATS’ was used for basic statistical analysis, ‘RMS’ for restricted cubic spline regression, and ‘SEGMENTED’ for two-piecewise logistic regression. Statistical significance was determined using a threshold of *p* < 0.05 for two-tailed tests.

## 3 Results

### 3.1 Characteristics of subjects stratified by BMI tertiles at baseline

Table 1 presents the baseline characteristics of the 2,942 participants stratified by BMI tertiles. There were notable variations across the various BMI groups in terms of lifestyle, clinical factors, and demographics. Age showed a significant inverse trend across the tertiles (*p* < 0.001): subjects with the highest tertile had the lowest mean age (65.97 ± 5.07 years). Gender distribution varied significantly (*p* < 0.001): the proportion of females increased from 32.82% in the lowest tertile to 51.27% in the highest tertile. Marital status also differed significantly among different BMI groups (*p* = 0.030): the proportion of married individuals (86.03%) was highest in the subjects with the highest BMI tertile. While education levels were comparable among groups (*p* = 0.057), residence regions varied significantly (*p* < 0.001): a greater proportion of urban residents had higher BMI tertiles. Lifestyle factors such as smoking and drinking exhibited significant decreasing trends across the BMI tertiles (both *p* < 0.001). The probability of developing diabetes, hypertension, and dyslipidemia was significantly increased in the BMI group (both *p* < 0.001). Specifically, the incidence of diabetes rose from 9.68% in the lowest BMI tertile to 23.65% in the highest BMI tertile. Anthropometric and clinical parameters showed significant differences among BMI groups. SBP, DBP, FBG, TC, LDL-C, and TG increased significantly in higher BMI tertile groups (all *p* < 0.001). Conversely, HDL-C decreased markedly across the tertiles (*p* < 0.001). Blood creatinine level was comparable among different BMI groups (*p* = 0.485).

**Table 1:** Baseline characteristics of all participants.

Variables	Total	BMI tertile, kg/m^2^			*p*-value
		T1 (14.22–22.06)	T2 (22.07–25.10)	T3 (25.11–41.41)	
*N*	2,942	981	980	981	
Age, year	66.58 ± 5.35	67.53 ± 5.83	66.23 ± 4.98	65.97 ± 5.07	<0.001
Sex					<0.001
Male	1,688 (57.38%)	659 (67.18%)	551 (56.22%)	478 (48.73%)	
Female	1,254 (42.62%)	322 (32.82%)	429 (43.78%)	503 (51.27%)	
Marital status					0.030
Non-married	486 (16.52%)	173 (17.64%)	176 (17.96%)	137 (13.97%)	
Married	2,456 (83.48%)	808 (82.36%)	804 (82.04%)	844 (86.03%)	
Education					0.057
No formal education	1,084 (38.00%)	380 (39.75%)	339 (35.80%)	365 (38.42%)	
Primary school	856 (30.00%)	306 (32.01%)	285 (30.10%)	265 (27.89%)	
Middle school	847 (29.69%)	253 (26.46%)	301 (31.78%)	293 (30.84%)	
College or above	66 (2.31%)	17 (1.78%)	22 (2.32%)	27 (2.84%)	
Residence					<0.001
Urban	1,265 (43.00%)	322 (32.82%)	435 (44.39%)	508 (51.78%)	
Rural	1,677 (57.00%)	659 (67.18%)	545 (55.61%)	473 (48.22%)	
Smoking					<0.001
No	2,061 (70.08%)	565 (57.59%)	714 (72.93%)	782 (79.71%)	
Yes	880 (29.92%)	416 (42.41%)	265 (27.07%)	199 (20.29%)	
Drinking					<0.001
No	1,803 (61.31%)	550 (56.12%)	590 (60.20%)	663 (67.58%)	
Yes	1,138 (38.69%)	430 (43.88%)	390 (39.80%)	318 (32.42%)	
DM					<0.001
No	2,438 (82.87%)	886 (90.32%)	803 (81.94%)	749 (76.35%)	
Yes	504 (17.13%)	95 (9.68%)	177 (18.06%)	232 (23.65%)	
Dyslipidemia					<0.001
No	2,136 (72.60%)	824 (84.00%)	699 (71.33%)	613 (62.49%)	
Yes	806 (27.40%)	157 (16.00%)	281 (28.67%)	368 (37.51%)	
Hypertension					<0.001
No	1,922 (66.05%)	736 (75.64%)	635 (65.20%)	551 (57.22%)	
Yes	988 (33.95%)	237 (24.36%)	339 (34.80%)	412 (42.78%)	
SBP, mmHg	131.85 ± 19.85	126.44 ± 19.76	133.07 ± 19.91	136.05 ± 18.65	<0.001
DBP, mmHg	75.57 ± 11.19	72.14 ± 10.66	76.52 ± 11.21	78.06 ± 10.85	<0.001
FBG, mg/dl	102.00 ± 29.35	96.18 ± 24.06	103.28 ± 30.87	106.54 ± 31.60	<0.001
Creatinine, mg/dl	0.85 ± 0.33	0.85 ± 0.24	0.85 ± 0.45	0.84 ± 0.27	0.485
TC, mg/dl	184.98 ± 36.09	179.75 ± 35.43	187.66 ± 35.98	187.54 ± 36.33	<0.001
HDL-c, mg/dl	51.24 ± 11.74	55.31 ± 12.75	50.50 ± 11.29	47.90 ± 9.77	<0.001
LDL-c, mg/dl	103.86 ± 28.83	99.94 ± 28.46	105.85 ± 28.19	105.77 ± 29.46	<0.001
TG, mg/dl	134.27 ± 82.51	103.61 ± 58.92	139.10 ± 86.38	160.11 ± 88.74	<0.001

The variables are presented as *n* (%) or the mean ± SD; BMI, body mass index; DBP, diastolic blood pressure; DM, diabetes mellitus; FBG, fasting blood glucose; HDL-c, high-density lipoprotein cholesterol; LDL-c, low-density lipoprotein cholesterol; SBP, systolic blood pressure; TC, total cholesterol; TG, triglyceride.

### 3.2 BMI and cognitive impairment in multivariate logistic regression analysis

During the 3-year follow-up, 600 participants (20.4%) experienced cognitive impairment. Table 2 presents the correlation between BMI and cognitive impairment across three models. In all models, an inverse relationship was noted between BMI as a continuous variable and cognitive impairment. In the Model II, the odds of cognitive impairment reduced by 4% for every unit rise in BMI (OR: 0.96, 95% CI: 0.93–0.99, *p* = 0.008). This relationship remained consistent across all models, suggesting a robust association between higher BMI and lower risk of cognitive impairment. When analyzing BMI by tertiles, a dose–response relationship became apparent. In the Model II, as compared to the subjects with the lowest BMI tertile (T1), subjects with middle BMI tertile (T2) showed a non-significant reduction in cognitive impairment risk (OR: 0.84, 95% CI: 0.66–1.06, *p* = 0.136). Conversely, the highest BMI tertile (T3) demonstrated an evident protective effect (OR: 0.64, 95% CI: 0.50–0.83, *p* < 0.001). The trend analysis across tertiles was statistically significant in both Model I (*p* = 0.013 for trend) and Model II (*p* = 0.009 for trend), indicating a progressive decrease in cognitive impairment risk with increasing BMI.

**Table 2:** Relationship between BMI and cognitive decline in different models.

Variable	*N*	Unadjusted Model		Model I		Model II	
		OR (95%CI)	*P*-value	OR (95%CI)	*P*-value	OR (95%CI)	*P*-value
BMI, kg/m^2^	2,942	0.96 (0.94, 0.98)	< 0.001	0.96 (0.94, 0.99)	0.007	0.96 (0.93, 0.99)	0.008
BMI tertile							
T1 (13.93–22.22)	981	Reference		Reference		Reference	
T2 (22.23–25.40)	980	0.77 (0.63, 0.94)	0.010	0.85 (0.68, 1.06)	0.157	0.84 (0.66, 1.06)	0.136
T3 (25.41–47.89)	981	0.64 (0.52, 0.78)	< 0.001	0.68 (0.54, 0.85)	0.001	0.64 (0.50, 0.83)	< 0.001
*p* for trend		0.053		0.013		0.009	

OR, odds ratio; CI, confidence interval; Unadjusted Model adjusted for none; Model I adjusted for age; sex; marital status; residence; Model II adjusted for sex; age; marital status; residence; smoking; drinking; hypertension; DM; dyslipidemia; creatinine; TC; HD; LDL.

### 3.3 Non-linear connection between BMI and cognitive impairment

**Figure 2:**
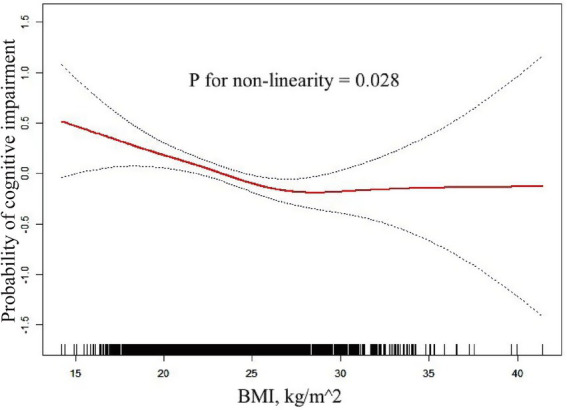
Non-linear connection between BMI and cognitive impairment. The completely adjusted smooth curve fitting demonstrated a nonlinear connection between BMI and Cognitive Impairment (*p* = 0.028 for nonlinearity).

The completely adjusted smooth curve fitting (Figure 2) demonstrated a nonlinear connection between BMI and cognitive impairment (*p* = 0.028 for nonlinearity). As shown in Table 3, this nonlinear relationship was further analyzed using a two-piecewise logistic regression model. Results showed a significant inflection point at BMI of 26.60 kg/m^2^, indicating a change in the nature of association at this threshold. For BMI lower than 26.60 kg/m^2^ (*n* = 2,342), an evident inverse relationship was noted between BMI and cognitive impairment (OR: 0.96, 95% CI: 0.93 to 0.99, *p* = 0.008). The analysis revealed that the odds of cognitive impairment decreased by 4% per unit increase in BMI up to 26.60 kg/m^2^. Beyond this threshold (BMI ≥ 26.60 kg/m^2^, *n* = 600), no significant relationship was found between BMI and cognitive impairment (OR: 1.04, 95% CI: 0.97–1.12, *p* = 0.312). Two slopes were significantly different (OR: 1.11, 95% CI: 1.01 to 1.22, *p* = 0.027), indicating a meaningful change in the relationship at the inflection point.

**Table 3:** The results of two-piecewise logistic regression model.

Inflection points of BMI	OR	95%CI	*P*-value
Inflection point	26.60		
< 26.60 slope 1 (*n* = 2,342)	0.96	0.93 to 0.99	0.008
≥26.60 slope 2 (*n* = 600)	1.04	0.97 to 1.12	0.312
Slope 2 – slope 1	1.11	1.01 to 1.22	0.027
Predicted at 26.60	−1.32	−1.48 to −1.16	
Log likelihood ratio test			0.028

Effect: cognitive impairment in 2018; Cause: BMI, adjusted for sex, age, marital status, residence, smoking, drinking, hypertension, DM, dyslipidemia, creatinine, TC, HD, LDL. Slope 1 and slope 2 represent the slope coefficients for the segments preceding and following the inflection point.

## 4 Discussion

This research represents an extensive longitudinal analysis to examine the link between BMI and cognitive impairment the older adult population aged 60 and above in China, utilizing the CHARLS dataset from 2015 to 2018. The important findings of our study were: (1) In Chinese older adult population, those with higher BMI faced a reduced risk of cognitive impairment: each unit increase in BMI corresponded to a 4% decrease in the odds of cognitive impairment; (2) Compared with the individuals in the lowest BMI tertile group, individuals in the highest BMI tertile group had a 36% lower risk of cognitive impairment; (3) The link between BMI and cognitive impairment was shown to be non-linear, with an inflection point at 26.60 kg/m^2^. A significant inverse relationship was noted when the BMI was lower than 26.60 kg/m^2^, and no significant association was found when the BMI was higher than 26.60 kg/m^2^. These findings support the existence of the “obesity paradox” in cognitive function among older adult Chinese adults, particularly in those who are overweight, and highlight a complex, non-linear correlation between BMI and cognitive health within this population.

The current study identified a negative link between BMI and cognitive impairment, providing further support for the “obesity paradox” in cognitive function among older adult adults. This paradox refers to the counterintuitive finding that being overweight or mildly obese may confer protective effects against certain health outcomes, including cognitive impairment, in older adult Chinese population (Lavie et al., 2015; Liang et al., 2022). Our results were consistent with previously reported. Maharan et al. reported that obesity was positively related to cognition (Maharani and Tampubolon, 2016). Sobow et al. (2014) reported that older adult adults with a low BMI (<25 kg/m^2^) and those who were losing weight had higher risk of dementia and faster cognitive impairment. Investigators have proposed several mechanisms to explain this paradox. Obesity or higher BMI in older adult adults mainly attribute to the accumulation of fat in the legs (Kim et al., 2016). Increased leg fat mass in older adult adults is associated with improvement of glucose metabolism (Snijder et al., 2004), ultimately reducing the risk of cognitive impairment (Banks et al., 2012). Higher BMI in older adult adults indicates better nutritional status and energy reserves, which is particularly important in older adult adults with a risk of malnutrition or frailty. Adequate nutrition, including sufficient intake of essential nutrients and calories, is crucial to maintain cognitive function and may help buffer against age-related cognitive impairment (Dauncey, 2009). Adipose tissues, which increase with higher BMI, are not only an inert storage depot but an active endocrine organ. They can produce leptin, a hormone that has been shown to exert neuroprotective effects and may improve cognitive function (Luchsinger and Gustafson, 2009). Leptin has been found to promote neurogenesis, reduce neurodegeneration, and improve synaptic plasticity, all of which are crucial to maintain cognitive health (Malekizadeh et al., 2017). The hippocampus is important for learning and memory, and leptin may modulate the function of the hippocampus, which potentially explains its protective effects on cognitive function (Farr et al., 2006). Additionally, higher BMI is associated with increased estrogen level in postmenopausal women, which may have cognitive benefits (Yaffe et al., 2000). Estrogen has been shown to possess neuroprotective properties, including reducing oxidative stress, promoting neuronal survival, and improving neurotransmitter function (Arevalo et al., 2015). These effects are helpful to preserve cognitive function in older adult women with higher BMI. Furthermore, individuals with higher BMI may have greater cognitive reserve due to increased brain volume, which potentially delays the cognitive impairment (Katzman et al., 1988). Cognitive reserve refers to the ability of the brain to deal with damage and maintain function, and it is related to some factors such as education, occupation, and brain size. The increased brain volume associated with higher BMI may provide additional neural resources that can be recruited to maintain cognitive function in case of age-related brain changes (Stern, 2012).

However, a non-linear relationship between BMI and cognitive impairment was discovered in the present study, with an inflection point at a BMI of 26.60 kg/m^2^, indicating a more complex interaction between BMI and cognitive function. This non-linearity may reflect the balance between the protective effects of higher BMI and the detrimental effects of extreme obesity. Below the inflection point, the protective effects of higher BMI are dominant, while above this inflection point, the negative impacts of obesity, such as increased inflammation and vascular dysfunction, may compromise these benefits (Miller and Spencer, 2014). The inflammatory processes associated with obesity play a crucial role in this balance. While moderate increase in BMI may provide benefits discussed above, extreme obesity may lead to chronic mild inflammation, which is closely related to cognitive impairment and neurodegenerative diseases (Sochocka et al., 2019). Pro-inflammatory cytokines produced by excess adipose tissues can cross the blood–brain barrier and negatively impact neuronal function and survival (Miller and Spencer, 2014). Moreover, obesity-related vascular dysfunction may contribute to cognitive impairment in cases of extreme obesity. Obesity may increase the propensity for hypertension, diabetes, and cardiovascular conditions, all of which can impair cerebral blood flow, leading to cognitive impairment (Gorelick et al., 2011). The compromised vascular health as a consequence to extreme obesity may adversely affect the higher BMI related protective effects on the cognitive function. This finding underscores the importance of considering BMI as a continuous variable and exploring potential non-linear relationships in future studies of cognitive health in older adult adults. Our results indicate that there may be an optimal BMI range for cognitive health in older adult adults, beyond which the risks begin to outweigh the benefits. In the future, studies should focus on the identification of this optimal range and the investigations of mechanisms driving the shift from protective to detrimental effects of increased BMI on the cognitive function.

This study has several strengths. First, the source of the data was CHARLS, a large-scale, nationally representative longitudinal study that improves the generalizability of our findings to the older adult Chinese population by offering a strong sample size. Second, the longitudinal design enabled the assessment of the temporal link between BMI and cognitive impairment, offering stronger evidence for causality compared to cross-sectional studies. Third, we conducted comprehensive statistical analyses, incorporating both linear and non-linear models, to better characterize the complicated linkage between BMI and cognitive impairment. The non-linear relationship at an inflection point deepens our understanding of BMI-cognition association in older adult adults. However, there were still limitations in the present study. First, while MMSE is widely used to assess cognitive impairment, it may not capture subtle changes in specific cognitive domains. Thus, more comprehensive neuropsychological assessments may be employed in future studies. Second, residual confounding factors unmeasured cannot be excluded despite adjusting for multiple confounders. For example, information about dietary pattern, physical activity level, and genetic factor, was unclear in this population and these factors may also affect both BMI and cognitive function. Third, even though BMI is a measure of adiposity, it is unable to distinguish between lean and fat mass. Alternative body composition metrics (such as body fat percentage and waist circumference) may shed light on the association between adiposity and cognitive impairment. Fourth, the follow-up period was too short (just three years), leaving insufficient time to capture long-term cognitive changes. Therefore, more studies with longer follow-up period may provide more important evidence on the relationship between cognitive impairment and BMI in older adult adults.

In conclusion, this longitudinal study provides evidence supporting the “obesity paradox” in cognitive function among Chinese older adult people. A non-linear association exists between BMI and cognitive impairment with BMI at an inflection point, and higher BMI correlates with a reduced probability of cognitive impairment, particularly in overweight subjects. These outcomes highlight a multifaceted connection between older adult persons’ BMI and cognitive health, underscoring the necessity for sophisticated weight-management techniques in this demographic. Our analysis’s inflection point suggests that the beneficial impact of increased BMI on cognitive health may have boundaries. To clarify the reasons behind this contradictory association and determine the ideal BMI range for older adult persons’ cognitive health, more research is necessary. Our findings emphasize the importance of age-specific BMI recommendations and indicate that maintaining a slightly higher BMI in late life may benefit the cognitive health. However, given the known health risks associated with obesity, these findings should be interpreted cautiously, and individualized approaches are warranted to weight management in older adult adults. Further longitudinal studies with longer follow-up period and more comprehensive measures of body composition and cognitive function are needed to certify our findings.

## Data Availability

The datasets presented in this study can be found in online repositories. The names of the repository/repositories and accession number(s) can be found at: http://charls.pku.edu.cn/en.

## Footnotes

^1^ http://charls.pku.edu.cn/en
